# A Series-Based Deep Learning Approach to Lung Nodule Image Classification

**DOI:** 10.3390/cancers15030843

**Published:** 2023-01-30

**Authors:** Mehmet Ali Balcı, Larissa M. Batrancea, Ömer Akgüller, Anca Nichita

**Affiliations:** 1 Faculty of Science, Mathematics Department, Muğla Sıtkı Koçman University, 48000 Muğla, Turkey; 2 Department of Business, Babeş-Bolyai University, 400174 Cluj-Napoca, Romania; 3 Faculty of Economics, “1 Decembrie 1918” University of Alba Iulia, 510009 Alba Iulia, Romania

**Keywords:** 4D classification, deep learning, lung nodule image, radial scanning

## Abstract

**Simple Summary:**

Medical image classification is an important task in computer-aided diagnosis, medical image acquisition, and mining. Although deep learning has been shown to outperform traditional methods based on handcrafted features, it remains difficult due to significant intra-class variation and inter-class similarity caused by the diversity of imaging modalities and clinical pathologies. This study presents an innovative method that is an intersection between 3D image analysis and series classification problems. Therefore, the self-similarity features in medical images are captured by converting the regions of interest to series with a radial scan and these series are classified with U-shape convolutional neural networks. The findings of this study are expected to be used by researchers from various disciplines working on radial scanned images, as well as researchers working on artificial intelligence in health.

**Abstract:**

Although many studies have shown that deep learning approaches yield better results than traditional methods based on manual features, CADs methods still have several limitations. These are due to the diversity in imaging modalities and clinical pathologies. This diversity creates difficulties because of variation and similarities between classes. In this context, the new approach from our study is a hybrid method that performs classifications using both medical image analysis and radial scanning series features. Hence, the areas of interest obtained from images are subjected to a radial scan, with their centers as poles, in order to obtain series. A U-shape convolutional neural network model is then used for the 4D data classification problem. We therefore present a novel approach to the classification of 4D data obtained from lung nodule images. With radial scanning, the eigenvalue of nodule images is captured, and a powerful classification is performed. According to our results, an accuracy of 92.84% was obtained and much more efficient classification scores resulted as compared to recent classifiers.

## 1. Introduction

Cancer is one of today’s most serious health issues. Despite significant and promising advances in medicine, the desired level of prevention and elimination of many cancers has yet to be achieved [1,2,3]. Cancer is a common disease that is difficult, time-consuming, and challenging to treat. It is diverse with numerous subtypes. Some types of cancer, which are common in most people, are lethal. Cancer treatment is a difficult process, and early detection is critical. Early cancer diagnosis can be helped by a clinical follow-up of the patient in later stages. In this context, screening is the search for the presence of cancer cells in humans who have no symptoms. Screening stages are the most important steps in the fight against cancer because they are required for early diagnosis. Information obtained by imaging methods is used to determine the cancer type and its stage, which are extremely useful for disease treatment planning. As a result, the accuracy of information obtained by scanning methods can change the outcome of the disease. Patients can live a longer and more fulfilling life due to correct screening methods and treatment plans that are determined in conjunction with accurate analyses. The application of advanced technology in cancer imaging, which is required for a patient’s treatment plan, as well as correct evaluation, are highly effective for determining treatment plans. Patients who have the opportunity to benefit from proper imaging techniques gain an advantage during the difficult treatment process by correctly analyzing imaging data.

Due to the high cost of equipment and personnel, as well as the difficulty of the task, it is not possible to apply known screening programs to every person. Lung nodules come in a wide range of shapes and sizes, hence identifying and characterizing abnormalities in these nodules is a difficult and delicate task. In this regard, computer-aided diagnosis (CAD) systems are critical to make clinicians’ jobs easier.

Image processing and machine learning-based research on digital pathology image classification have yielded promising results. These findings suggest that digital pathology systems based on machine learning could be widely used in pathology clinics. Artificial intelligence and machine learning-based solutions will be used at a much higher rate in the coming years, particularly in pathology.

The mortality rate from lung cancer is the greatest of any kind of cancer, although this is a disease whose prognosis may be improved with early diagnosis. In order to establish which pulmonary nodules are benign and which nodules need biopsy to confirm malignancy, low-dose computed tomography has become the standard procedure for lung cancer screening. Nevertheless, lung cancer screening has a significant clinical false-positive rate because of the necessity to identify a high proportion of malignant nodules for biopsy [4,5]. Due to this, many unnecessary biopsies are conducted on people who turn out not to have cancer.

In this study, we provide a CNN architecture that combines data from volumetric radiomics series and nodule images for categorization. Qualitative and quantitative characteristics may be found in lung CT images. These characteristics illustrate the nodule’s pathogenesis. Using mathematics and data characterization methods, these quantitative characteristics are retrieved from the picture. The term “radiomic” is used to describe the procedure, whereas “radiomic features” refers to the numerical characteristics that are gleaned from the data. As defined in [6], this process involves “high-throughput extraction of quantitative information from radiological pictures to build a radiomic, high-dimensional dataset followed by data mining for possibly better decision support.” The radiomic characteristics of nodules primarily include their morphology, shape, and gray-level distribution. This research uses a spherical radial scan of a 3D model derived from CT scans to decode information about the nodule’s volume and shape over time. The created regions in each level plane are scanned radially while the planes themselves are shifted from bottom to top. Thus, the shape shift may be considered with the gray level distributions of the CT scans collected at the various stages. Using the LIDC-IDRI dataset, we take a novel method to predict the malignancy of lung nodules by integrating hitherto unexplored image and volumetric radiomic combinations with volumetric radiomics-induced series.

CAD methods still have several limitations, despite numerous studies demonstrating that deep learning approaches outperform traditional methods based on manual features [7,8,9,10]. This is due to the fact that imaging modalities and clinical pathologies differ. Such diversity creates difficulties because of differences and similarities between classes. In this context, the new approach in our study is a hybrid method that classifies data using both medical image analysis and series features. Image-derived interest areas are subjected to a radial scan, with their centers acting as poles, in order to obtain series. A convolutional neural network (CNN) model is used to solve the series classification problem. We advance a method for classifying series obtained from lung nodule images. The eigenvalue of the nodule images is captured using radial scanning and a powerful classification is performed. According to our results, we obtained an accuracy of 92.44% and significantly higher classification scores as compared to numerous traditional classifiers.

### Related Works

Many pre-diagnosis models capitalize the advantage of CNN architectures that revolutionized computer vision research by making color images usable as input data. In this context, input data are processed by a succession of cores that slide over image color channels to extract characteristics such as edges and color gradients, giving the appearance of an artificial neural network’s (ANN) downstream fully linked layers. These inputs are summed and flattened before being sent on to the fully linked layer. Several different kinds of preconfigured CNN architectures are available. Radiology and digital pathology both benefit greatly from the usage of CNNs.

There has been extensive research into the development of CAD systems for lung cancer screening. Detection and segmentation of pulmonary nodules, characterization of nodules, and classification of malignancy were among the studies that stood out. Recently, very good and promising results in lung cancer screening, as well as other cancer screenings, have been obtained, with deep learning-supported studies on nodule detection, segmentation, and characterization [11,12,13].

Capabilities of CADs and radiomic tools to improve diagnostic accuracy and consistency across medical images help radiologists’ decision-making [14,15]. CADs and radiomics rely on segmentation and quantitative feature extraction from images of identified nodules as its foundation. Moreover, machine learning algorithms use this collection of properties as a training set for classifying unseen nodule samples [16,17]. Such studies focus on the intranodular region and employ radiomic characteristics of its shape, boundary, and tissue for the most accurate identification [18,19,20,21,22].

Deep learning saves time for medical professionals by performing the complex classification task, which requires a significant amount of time and effort and consists of the classification of large amounts of images, while avoiding possible human-induced lines during the diagnosis phase at the same time [23,24,25]. Although it is well known that accurate and early diagnosis are effective in all disease types, deep learning-based methods have been successfully applied in early diagnosis, which is a crucial stage in cancer disease [26,27,28]. Deep network architectures have evolved and their computational power has increased as deep learning models have advanced in specific tasks. Deep neural networks have begun to be used effectively in computer vision processes such as image classification, object detection, and image segmentation as CNNs have made significant progress. Deep learning and CNN advancements have been critical in the development of medical systems for reliable scanning and image-based diagnostics. As a result, research has progressed from image segmentation and feature extraction to deep learning-based automatic classification [29,30].

Abdoulaye et al. [31] classified mammography images into three stages. First, they removed noise from the image by examining its surroundings, then they discovered the physical properties of the object and extracted patterns. In this way, they were able to create a cancer detection system based on the artificial intelligence-enabled algorithm that they trained using a pattern they obtained. Wang et al. [32] used an automatic image analysis technique to classify breast cancer histopathology images. They obtained 4 shape-based features and 138 color-space features for nodule classification. As a preprocessing step, they used bottom-up cap transformation to highlight background objects in order to locate growing cancer cells. Afterwards, they used wavelet transform to determine the location of ROIs, and as a result, they classified normal and malignant cell images with a 96.19% success rate. Jiang et al. [33] developed their own method by studying lymphatic pathologies such as chronic lymphocytic leukemia (CLL), follicular lymphoma (FL), and mantle cell lymphoma (MCL). After preprocessing the image, they extracted a feature set that included texture properties such as entropy, density mean, density standard deviation, loopy back propagation, and gray level co-occurrence matrix. They used the support vector machine (SVM) algorithm to classify pathology images based on the extracted features. As a result, their average accuracy performance value was 97.96%. Mohammed et al. [34] trained ANNs to predict pancreatic cancer risk using clinical variables such as age, smoking status, alcohol consumption, and ethnicity.

Busnatu et al. [35] and Hunter et al. [36] present a detailed account of the recent literature studies on artificial intelligence and deep learning applications classified according to medical specialties. Readers can refer to these two studies for more comprehensive information on deep learning applications regarding cancer diagnosis based on image analysis.

Image series can be created by taking temporary images of the same scene at different ordered input. If each sequential input corresponds to the time tick, it is possible to say that the obtained series are time series. Several researchers have developed effective methods for correctly interpreting image time-series data as a result of acquiring image data [37,38,39,40,41,42,43,44,45,46,47]. With early diagnosis and a correct treatment, the quality of patients’ lives can be substantially improved due to the analysis of biomedical time series via accurate and reliable techniques, the understanding of such data, and the rapid detection of possible abnormalities. The use of temporal correlation in time-series analysis is critical to the success of chosen methods. In this context, image time series are critical in biomedicine for monitoring disease progression.

Iakovidis et al. [37] used time series obtained from chest radiographs to track the progression of pneumonia. Contrariwise, Baur et al. [38] used canonical correlation analysis and Dynamic Bayesian Networks (DBN) to extract validated gene regulatory networks from time-series gene expression data. Likewise, Guo et al. [39] built gene regulatory networks with a feature selection algorithm based on partial least squares (PLS). In their studies, Penfold et al. [40] and Isci et al. [41] introduced Bayesian methodologies for network analysis using biological data, especially measures of time-series gene expression. Schlitt et al. [42] used Bayes and DBNs to explain gene expression variations over time in terms of regulatory network topologies. According to Ni et al. [43] and Kim et al. [44], the study of Murphy et al. [45] suggested techniques capable of expressing time-varying behavior of the underlying biological network, hence offering a more accurate representation of spatio-temporal input–output connections. In their work, Kourou et al. [46] used time-series microarray gene expression data to classify differentially expressed genes (DEGs) in cancer with great effectiveness. Imani et al. [47] expanded the analysis of radio frequency (RF) time series to enhance tissue classification at clinical frequencies by using additional time-series spectrum characteristics.

Various non-local deep learning architectures, which we also used in the comparison analysis, have been successfully used in the nodule classification task. Shen et al. [48] proposed multi-crop convolutional neural networks and Al-Shabi et al. [49] advanced gated-dilated networks for malignancy classification and obtained above 87% accuracy scores. Moreover, Ren et al. [50] built a unique manifold regularized classification deep neural network (MRC-DNN) to conduct classification directly based on the manifold representation of lung nodule images, which was motivated by the observation that genuine structure among data was typically contained on a low-dimensional manifold. Shen et al. [51] showed that the resilience of a representative DL-based lung-nodule classification model for CT images could be improved, highlighting the need of assessing and assuring model robustness while creating comparable models. To increase the depth of representation, Jiang et al. [52] first developed a contextual attention mechanism to model contextual relationships between neighboring sites. Then, authors employed a spatial attention technique to automatically find the zones that were crucial for nodule categorization. Finally, they used an ensemble of models to increase the reliability of their predictions. Al-Shabi et al. [53] suggested using residual blocks for local feature extraction and non-local blocks for global feature extraction. Furthermore, Al-Shabi et al. [54] used 3D Axial-Attention, which only needs a little amount of processing power as compared to a traditional non-local network.

## 2. Methodology

The 3D volumetric structure comprises the sections designated as nodules by radiologists from 2D CT scans, together with the series derived from the boundary curves of each section. The following paragraphs explain boundary curves and the process of extracting series out of them. Moreover, details on 3D models and the underlying deep learning framework are provided.

### 2.1. Series by Radial Scanning

A radial scan gathers image samples in a sparser distribution at the periphery of the image and in a denser distribution closer to the center of the image. This is the preferred scanning paradigm for several imaging applications, such as imaging the optic nerve head, as each B-scan acquired includes a cross-sectional image of the optic cup [55,56,57]. The volumetric, render, and morphometric analysis of the ensuing image may be used to see and analyze the radially obtained data samples. A straightforward radial-to-Cartesian coordinate translation may be used to resample data to a Cartesian mesh system.

**Figure 1:**
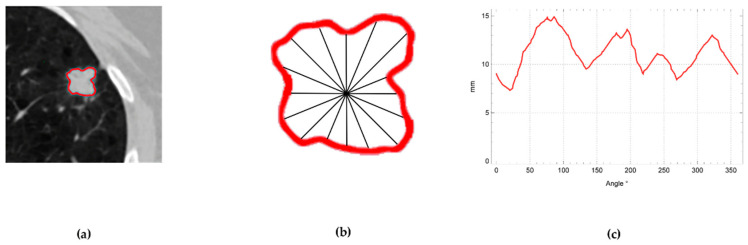
Method of obtaining series by radial scanning.

Figure 1 provides a radial scan as an illustration. The region of interest of a lung nodule imaging is shown in Figure 1a. The radial scan axis is positioned at the center of the area of interest, and the boundary curve of the area of interest is depicted in Figure 1b. The boundary curve points’ separation from the scanning center will vary as the scanning angle changes, resulting in a series, as illustrated in Figure 1c. 

ROIs are portions of a designated data collection that are used for a certain objective. The term ROI is often used in a variety of application fields. For instance, in medical imaging, the borders of a tumor can be specified in an image or a volume to determine its size. For the purpose of assessing cardiac function, the endocardial boundary can be seen on an image at various points in the cardiac cycle, such as end-systole and end-diastole. The ROI establishes the perimeters of an item under inspection in computer vision and optical character recognition.

The CT images used in this study first underwent pixel-by-pixel binarization. After this morphological processing, large components in the binarized images are handled as ROIs. The center of the ROI is used to calculate the discrete center of gravity of the ROI for radial scanning. Due to the binary nature of the image, this center may be easily located without any weight.

The modified Canny edge recognition approach [58] is first applied to the ROI in each image to extract the appropriate form attributes. This extraction is made possible via the use of the improved Canny edge detector approach (one for each ROI within each image). The Canny operator employs a multi-step process to identify the edge pixels of an object. The first step is to adjust the area boundaries by using a Gaussian filter. After that, a regular 2D first derivative operator is used to compute edge strength. Pixels that are not a component of the local maximum are zeroed out when the non-maximum suppression method scans the region in the gradient direction. Lastly, a threshold is employed in order to determine the correct edge pixels. Therefore, each ROI may be represented by its own border curve.

It is essential to streamline the edges for ROI representation while extracting image features. The aim of the region boundary simplification stage is to create a smooth curve while minimizing the number of line segments used to delineate the area. This method is known as polygon approximation, and it is used to approximate a polygon curve that has a set number of vertices. The polygon curve approach looks for a subset of the initial vertices in order to minimize the objective function. The min-number problem is only one way to frame the issue. The appropriate approximation of an N-corner polygon curve is achieved by joining a certain number of straight-line M segments with another polygon curve. A common heuristic for finding a solution to the minimum number problem is the Douglas–Peucker (DP) method [59].

In this study, prior to using the Hough transform to extract features, the borders of ROIs are simplified using the Douglas–Peucker (DP) technique. The closeness of a vertex to an edge segment is a factor in the DP method. This approach operates top-down, beginning with a rough initial estimate on a simplified polygonal curve, or more specifically, on the single edge linking the first and end vertices of the polygonal curve. Then we determined the closeness of the remaining vertices to that edge. The corner that is furthest from the edge is added to the simplification if there are vertices further away from the edge than the provided tolerance (ε>0). As a result, the reduced polygonal curve receives a new estimate. Recursion is used to continue this process for simplification until all vertices of the original polygonal curve fall inside the tolerance.

If the ROI border is considered a closed curve, we must figure out the optimal distribution of all neighboring vertices, including the initial one. The simplest approach is to start from the vertex with the fewest errors. Compared to the open-curve procedure, this simple method for a curve with N corners is N times more complicated to implement. There are a number of options to consider when deciding where to set off. This research makes use of a heuristic technique inspired by Sato’s strategy [60]. The first step in this procedure is starting at the furthest location from the ROI’s spatial center.

### 2.2. 3D Nodule Segmentation

In this research, computer-assisted techniques were used to identify nodules. Automatic nodule recognition and segmentation is achieved using the union of the You Only Look Once, Version 3 (YOLOv3) [61] and iW-Net [62] architectures. The short version is that the model is fine-tuned to identify lung nodules by minimizing a loss function that considers breadth, height, and center of gravity of the estimate in comparison to the baseline. In order to take 3D information into account, the algorithm is trained using 3-channel images that consist of one axial slice comprising the nodule center as well as two equally spaced neighboring slices. Candidates are joined if their bounding boxes overlap, and estimates are calculated for each axis slice. Only the first block of iW-Net, which makes a segmentation prediction, is utilized for actual segmentation. We employ an image classification method to identify nodules with a bounding box in order to facilitate the use of temporal statistical classification with the series collected from the image. This image was achieved by manually creating these marks. Each image of interest has different dimensions according to the series methodology used in this research. After the series has been normalized, this variation has no bearing on the categorization.

The LIDC-IDRI database contains thoracic CT images with highly annotated lesions for the purpose of detecting lung cancer. The series acquisition approach for the automatically segmented nodule outlined how to find the nodule border by drawing a closed curve around each nodule wherever it was present, beginning at the first pixel outside the lesion. CT scan findings are recorded in an XML file connected with each participant. Nodules in each XML file are grouped into one of three sizes based on their diameter. The locations of the nodules and their z coordinates are included in the data. With these coordinates, we were able to generate a box and mask in three dimensions that were centered on the annotated lung nodule sites and were a fixed size. Our experimental boxes are 32 pixels square and 32 slices thick. Nodule boundary curves in the sections are scanned radially in 5625-degree increments to conform to the 3D volumetric data format. By using a thickness of 32 for the slices, we may encode the nodule’s border geometry as a matrix of type 32×32.

**Figure 2:**
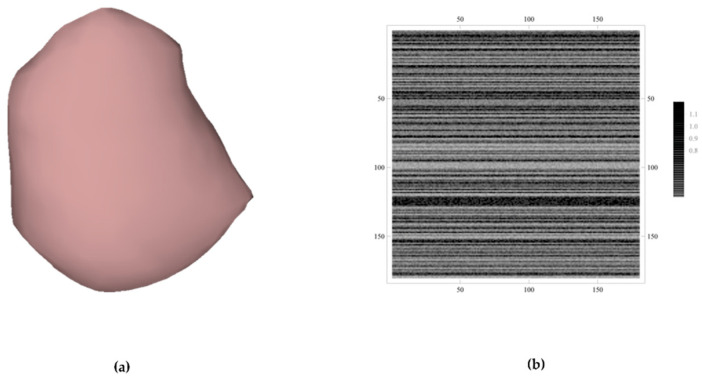
(**a**) 3D segmented and smoothed nodule and (**b**) a 180×180 matrix encoding the boundary shape of each slice.

Figure 2 depicts a 3D segmented nodule and the aforementioned shape matrix. In order to explain the methodology, we ran 2D radial scans with an angle increase of 2 degrees and applied Laplace smoothing to the Delaunay mesh that we had derived from the boundary points shown in Figure 2. Following the smoothing of the nodule surface, 180 *z*-axis steps were chosen.

### 2.3. Classification with U-Net

Two-dimensional conventional CNN designs typically layer-by-layer integrate raw input data with learnable filters. It may be built using several layers, each of which is trained to recognize a particular aspect of an image. Each training image is passed through a series of filters of increasing granularity, and the resulting convolutional image serves as input for the layer below it. An image filter may begin with basic characteristics such as brightness and edges and progress to more complicated characteristics that better characterize the item being filtered. This study proposes a technique that works well inside a deep learning framework using higher-order CNNs for effective feature learning of CT image data from unprocessed information. This is accomplished by stacking many convolutional layers in order to collect a wide variety of representative characteristics. By using convolutions and trainable filters with specific filter coefficients, we can link input and output neurons.

**Figure 3:**
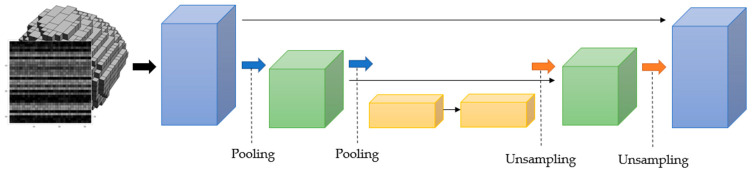
U-Net Architecture.

This paper provides a solution to the 4D input issue of jointly categorizing nodule volumetric radiomic and border information. For this challenge, we use a method centered on U-Net models that generalize 2D and 3D architectures [63,64]. As shown in Figure 3, we need to calculate the shape matrix obtained from radial scanning with a tensor that takes the coordinates in ${\mathrm{mm}}^{3}$ units of each volume segmented in the 3D volume and the grayscale value in these coordinates in order to train our 4D U-Net model efficiently and use it in the classification process. The model makes use of the 4D data input that it generates collectively. Lower-order models need data reduction prior to network training. In contrast, our suggested architecture makes extensive use of higher-dimensional data while performing all operations on nominally sized datasets.

The value of a convolved output neuron at coordinates $\left(k,l\right)$ in conventional 2D CNNs may be written as follows:

(1) $$y_{kl}=\varphi \left(\overset{C_{in}}{\underset{c}{\sum }}\overset{H-1}{\underset{i=0}{\sum }}\overset{W-1}{\underset{j=0}{\sum }}w_{ij}x_{c\left(k+i\right)\left(l+j\right)}-b_{ij}\right),$$

where $\varphi \left(\cdot \right)$ is the activation function, $w_{ij}$ is the value of the kernel connected to the current feature map at position $\left(i,j\right)$, $x_{c\left(k+i\right)\left(l+j\right)}$ is the value of the input neuron at input channel c, $b_{ij}$ is the bias of the computed feature map. Moreover, by following the extension method presented in [65], we can straightforwardly extend Equation (1) to 4D with

(2) $$y_{klmn}=\varphi \left(\overset{C_{in}}{\underset{c}{\sum }}\overset{R-1}{\underset{r=0}{\sum }}\overset{D-1}{\underset{d=0}{\sum }}\overset{H-1}{\underset{i=0}{\sum }}\overset{W-1}{\underset{j=0}{\sum }}w_{ijdr}x_{c\left(i:i+k\right)\left(j:j+1\right)\left(d:d+m\right)\left(r:r+n\right)}+b_{ijdr}\right).$$

With our deep pixel-level categorization, each pixel can only be assigned to one of C distinct categories. Because cross-entropy may be understood as the log-likelihood function of the training samples, it was chosen as the loss function to transform the network’s outputs back into probabilities. Training our models with this loss function combines the SoftMax activation with the cross-entropy loss to provide a probability across the C possible classes for each pixel.

## 3. Results

Overall, for this study, 244,559 images and 1018 CT scans from 1010 patients were provided by the Lung Image Database Consortium (LIDC) [66]. The five categories used to classify lesions in the LIDC image collection regarding pulmonary nodules are: highly likely to be benign (level 1); moderate probability of being benign (level 2); uncertain probability (level 3); moderate probability of malignancy (level 4); it is likely to be malignant (level 5). Due to the absence of a database structure, radiologists have not yet established relationships between images, examinations, and the possibility of malignancy from nodules, making the first LIDC image collection difficult to use. Thus, we choose to utilize the not only SQL (NoSQL) document-oriented Pulmonary Nodule Database (PND) [67] for our analysis.

The LIDC-IDRI study may be broken down into three major phases: image interpretation, nodule characteristic evaluation, and data recording. A radiologist was required to analyze each image of a CT examination using a computer interface and highlight lesions deemed to be nodules with in-plane dimensions between 3 and 30 mm, independent of assumed histology. As a result, these lesions may represent a primary lung cancer, a metastatic disease, a noncancerous condition, or of unknown etiology. Each nodule outline was intended to be a localizing “outside boundary” such that, according to the radiologist, the outline itself did not overlap nodule-specific pixels. According to the LIDC-IDRI literature, throughout the nodule characteristic evaluation procedure, each reader was requested to subjectively assign an integer value to nine distinct qualities. The data is stored in an eXtensible Markup Language (XML) file, and its classifications and Cartesian coordinates are based on nodule classifications. The XML file and all CT scans from a single test are kept in a folder, and all folders from all examinations were uploaded to a web server hosted on the website of the Cancer Imaging Archive (TCIA) [68]. In order to avoid unnecessary scans, PND only uses the radiologist’s annotations that identify the most lesions during each exam, which amounts to 752 scans and 1944 lung nodules. To normalize the image contrast, a gray-scale lung windowing was applied by adjusting the window/level from 1600 to −600 Hounsfield units.

Nodules, which may be up to 30 mm in diameter, are a kind of lung opacity [69]. Initially, we computed the nodule size as a straightforward 2D measure of the biggest diameter in a slice, which may be done in the axial plane along the axis of the longest diameter [70]. To get these rough estimates, we measured the *x* and *y* minimum and maximum coordinates of every nodule slice. According to [71], lung nodules with a PND malignancy grade of 3 were considered too dangerous to keep. We did not include any nodules in the LIDC collection that were annotated as non-solid because of the form complexity and low density of these objects. Therefore, following this phase, 897 nodules ranging in size from 3 mm to 30 mm remained (616 benign and 281 malignant). We were restricted from selecting smaller lesions due to the LIDC requirement of a 3 mm subthreshold.

A major restriction in this study was that the dataset has an uneven distribution of classes throughout its 897 nodules. During the phase of cross-validation training, the well-known Synthetic Minority Oversampling Approach (SMOTE) [72] method was used to develop synthetic nodule samples. This approach is also known as the synthetic minority oversampling approach. The method was developed with the intention of delivering a comprehensive and well-rounded approach. At each step of the process of cross-validation, nine folds were chosen to form the training set, whereas the remaining fold was used to form the test set. Moreover, we made sure that the appropriate proportions were preserved. Training sets comprised 550 benign nodules and 252 malignant nodules. Around 298 synthetic samples are produced by the SMOTE algorithm throughout each step of the procedure. This ensures that malignant nodules are represented as precisely as possible.

To assess the performance of the developed model, we employ a number of machine learning metrics, as the problem at hand is fundamentally a pixel-level multi-class classification task. True positives (TP), false positives (FP), false negatives (FN), and true negatives (TN) are the four possible outcomes when comparing a pixel’s prediction to its baseline accuracy score. True and False represent equality between the ground truth label and the predicted label, whereas Positive and Negative correspond to the class from which the metric is being calculated. In this study, common ML metrics are employed for each type of data using the above definitions. Namely, the metrics are:

(3) $$\mathrm{Recall}=\mathrm{TP}/\left(\mathrm{TP}+\mathrm{FN}\right)$$

(4) $$\mathrm{Precision}=\mathrm{TP}/\left(\mathrm{TP}+\mathrm{FP}\right)$$

(5) $$\mathrm{Accuracy}=\left(\mathrm{TP}+\mathrm{TN}\right)/\left(\mathrm{TP}+\mathrm{FP}+\mathrm{FN}+\mathrm{TN}\right)$$

(6) $$F1=2\times \left(\mathrm{Precision}\times \mathrm{Recall}\right)/\left(\mathrm{Precision}+\mathrm{Recall}\right)$$

The number of filters utilized for effective feature learning and the number of stack-layers in the proposed U-Net model are two major hyper-parameters that have a substantial impact on the model’s performance. In order to determine which combination of hyper-parameters produces the best results, we conducted an ablation study.

In the experiments, the effect of increasing the number of stack levels on the performance of the U-Net is analyzed. We trained two separate 4D U-Net models, one with a depth of 3 and the other with a depth of 4. Table 1 shows that the network’s generalization capacity increases when more filters are applied, suggesting that the network is becoming more robust. Naturally, the time needed to train the network grows in proportion to the number of filters, as each filter has its own set of parameters that must be learned. We also find that using only four filters in the U-Net, as opposed to eight, improves performance across the board when the depth is increased from three. Overall, the best U-Net model can be trained in around 11 h, has a depth of 3, and has a classification accuracy of 92.84%.

Table 2 summarizes and tabulates comparisons between our proposed method and state-of-the-art lung nodule classification methods. The results of our evaluations show that our proposed method consistently outperforms the state-of-the-art methods. Not only that, but it outperforms other non-local-based methods such as Local-Global [52], 3D Directed Partitioning Networks (DPNs) [53] and 3D Axial-Attention [54].

## 4. Discussion and Conclusions

Because lung nodules are so minuscule that they can easily blend in with the surrounding tissue and cling to complicated anatomical systems like the pleura, this work presents a deep learning strategy that additionally deals with volumetric radiomic information for classifying nodules in the lungs.

We started by obtaining 3-tensor data types representing gray levels of 3D nodule shape modeled from cross-sectional CT scans. Grayscale values between 0 and 255 are fed to this tensor at each node. Our study presents a deep learning classification solution to the age-old issue of picture classification by including the series collected from nodule segments. Our method takes into consideration the self-similarity of the boundary curves that characterize the nodule segments in order to provide a more precise categorization of nodules. By treating the series of the border curvatures of each section as rows in the matrix, we are able to solve the 4D classification issue.

For this research, we accessed a dataset hosted by LIDC. Over 95% accuracy was achieved when using the deep learning algorithms YOLOv3 and iW-Net to identify and isolate the nodules in the annotated photos. The respective photos were manually cropped and recorded in this LIDC dataset using tags. After that, we employed the image processing techniques described in the methodology section to locate the nodule’s outside and innermost curves. The use of 32×32-type matrices, the scanning at 5.625 degrees and 32 section steps yielded shape matrices that were consistent with the volumetric radiomics of the nodule.

The research used a U-Net-type convolutional neural network, which proved to be successful for the 4D categorization in previous studies [73,74,75,76]. Experiments were conducted using 4 and 8-filter meshes of depths 3 and 4, respectively. When compared to other networks, the one with three depths and eight filters performed quite well (92.84% accuracy). This outcome informed the selection of the network design from our study.

In the context of volumetric radiomics, comparisons were made between the results of this research and 3D CNN networks. The provided method yields superior performance results as compared to numerous non-native solutions presently available. The method presented in our research is most comparable to the 3D Axial Attention among the non-local approaches. Due to the fact that it takes into consideration the nodule’s 3D shape, the 3D Axial Attention approach is far more discriminating than earlier methods. However, the approach we present takes into account both the 3D geometry and the shape of the nodule, allowing for a 5D convolution. Although its performance is comparable to that of the 3D Axial-Attention approach, its outcomes are superior to those of prior methods. Future research may try using more radiomic variables within the framework of the 3D Axial Attention approach in order to get even more discriminating findings.

Limitations in the study design are inevitable, as is the case with every investigation. The primary barrier is the dearth of trained radiologists and experts in computer-assisted segmentation. The issue of class imbalance in the dataset may be addressed in a number of ways, all of which need careful consideration. Because the series angles derived from the radial-scanning boundary curve of the nodule follow one another in time, we may argue that the series represents a time series. An up-and-coming area of study in the field of forecasting is the use of time-series characteristics for model selection and model averaging [77,78,79]. However, most current methods need human intervention to choose a suitable collection of features. In modern time-series analysis, the use of machine learning techniques for automatically extracting characteristics from time series is becoming more important. Hybrid networks that can deal with radiomic features utilizing 3D geometry classification and machine learning-based time-series feature extraction may be studied in the future. Because our research demonstrates the usefulness of radial scanning, particularly in the context of medical image processing and classification, we believe it will serve as a benchmark for future studies examining other medical imaging methods.

In conclusion, we show that series from lung imaging may be used to effectively characterize lung nodules, and that a shape matrix, aided by an area of interest curve, can be used to reliably ascertain whether or not a tumor is malignant. We tested our methods using a large dataset of lung nodule pictures that was made accessible to the public, and we compared the outcomes to those produced by established methods for classifying both still photos and video over time. The requirement for our study to be repeatable prompted us to conduct these comparisons. Our research indicates that radial scanning series may be a powerful asset in the identification and categorization of lung nodules.

## Figures and Tables

**Table 1:** Metrics for classification and training times (in minutes) for 4D U-Net models.

Depth	No. of Filters	Recall	Precision	Accuracy	Time
3	4	80.13	81.54	83.45	469.92
	8	92.41	92.63	92.84	661.8
4	4	80.04	79.63	81.22	477.74
	8	87.19	88.01	88.73	668.4

**Table 2:** The proposed method’s performance compared to the state-of-the-art methods.

Method	AUC	Recall	Precision	Accuracy	F1
HSCNN [14]	85.6	70.5	N/A	84.2	N/A
Multi-Crop [48]	93.0	77.0	N/A	87.14	N/A
Local-Global [52]	95.62	88.66	87.38	88.46	88.01
Gated-Dilated [49]	95.14	92.21	91.85	92.57	92.03
3D DPN [53]	N/A	92.04	N/A	90.24	N/A
MRC-DNN [50]	N/A	81.00	N/A	90.00	N/A
Perturbated DNN [51]	91.0	90.0	N/A	83.0	N/A
3D Axial-Attention [54]	96.17	92.36	92.59	92.81	92.47
**Our method**	**96.19**	**92.41**	**92.63**	**92.84**	**92.51**

## Data Availability

The data presented in this study are available on request from the corresponding author.
